# The Status of Crime in 1859

**Published:** 1860-10-01

**Authors:** 


### Art. III.—THE STATUS OF CRIME IN 1859.

In the first number of this Journal for 1859,† we recounted briefly the history of Judicial Statistics in this country, and pointed out their great interest to the practical psychologist. At that time the able scheme of statistical registration, proposed by Mr. Samuel Redgrave, had only been partially carried out; now it has been fully achieved, and we design to gather from the last Report ‡ of that gentleman, such particulars as may serve to convey a notion of the status of crime in the kingdom, and of the operation of the chief means adopted for its suppression.

† See vol. ix., p. 75. ‡ Judicial Statistics, 1859. *Blue Book*, 1860.

1. *Police and Constabulary.*—The total number of police and constabulary in 1859, amounted to 20,597. Of this force, 6904 belonged to the metropolis. The proportion of police to resident population (at the best, Mr. Redgrave remarks, a very imperfect basis of comparing the relative amount of the police forces) averaged, throughout England and Wales, 1 in 870. In cities and towns, the proportion varies from 1 in 210 in the City of London, to 1 in 708 in Sheffield; in counties, from 1 in 1221 in Warwickshire, to 1 in 1602 in Suffolk. The cost of the police in 1859 amounted to 1,485,029*l.* 1*s.* 10*d.*, of which sum 310,205*l.* 10*s.* 7*d.* was contributed from the public revenue. The average expense of each man was 72*l.* 2*s.*—salaries and pay, 53*l.* 13*s.*; clothing and accoutrements, 5*l.* 1*s.* Of the repressive effects of an efficient police force, Mr. Redgrave thus observes:—

"The knowledge which a trained police obtains of all who live by unlawful pursuits—of their habits, associates, and modes of plunder, is of itself an important check; and the co-operation between the different bodies of police, which, though under the control of separate authorities, act upon the same system, and are united in the pursuit of offenders, renders the commission of crimes more hazardous, and the detection and conviction of offenders more certain. The effect of the extension and completion of the police system may be recognised in many of the results which appear in this (the first) part of the statistics; and the greater development and improvement which will arise from the experience of a large body of trained officers, must tend to the gradual repression of known habitual depredators. It will also prove a great check to professional mendicancy, particularly where the police are employed as relieving officers for vagrants, on the principle first adopted in the county of Essex, and since extended to many other districts—a due protection on the refusal of relief being found in the report of all cases to the Board of Guardians and the Court of Quarter Sessions." (p. v.)

2. *The Criminal Classes.*—In 1858, the police was instructed to ascertain and report the number of thieves, prostitutes, and suspected persons of all classes at large in England, and "for the first time their numbers were given as an ascertained fact, in opposition to the many estimates, chiefly of an exaggerated nature, which had from time to time been made." A like inquiry was instituted in 1859, and Mr. Redgrave observes that "it is a strong corroboration of the accuracy of the information possessed by the police, that, though very great differences appear on a comparison of the returns of the two years in the separate districts, as might be anticipated with regard to a body so largely migratory, yet the general total corresponds with peculiar exactness, as is shown by the following table:"—

*Numbers of the Criminal Class.*

| Classes. | 1859. | | | 1858. | | |
|---|---|---|---|---|---|---|
| | Males. | Females | Total. | Males. | Females | Total. |
| Known Thieves and Depredators: | | | | | | |
| Under 16 years of age... ... ... ... | 4,382 | 1,546 | 5,928 | 4,773 | 1,608 | 6,381 |
| 16 years and above ... ... ... ... | 26,478 | 7,132 | 33,610 | 26,772 | 6,879 | 33,651 |
| Receivers of Stolen Goods: | | | | | | |
| Under 16 years of age... ... ... ... | 85 | 28 | 113 | 119 | 29 | 148 |
| 16 years and above ... ... ... ... | 3,450 | 844 | 4,294 | 3,410 | 787 | 4,197 |
| Prostitutes: | | | | | | |
| Under 16 years of age... ... ... ... | — | 2,037 | 2,037 | — | 1,647 | 1,647 |
| 16 years and above ... ... ... ... | — | 28,743 | 28,743 | — | 27,113 | 27,113 |
| Suspected persons: | | | | | | |
| Under 16 years of age... ... ... ... | 3,878 | 1,370 | 5,248 | 3,912 | 1,512 | 5,424 |
| 16 years and above ... ... ... ... | 26,706 | 5,734 | 32,440 | 28,028 | 5,774 | 33,802 |
| Vagrants and Tramps: | | | | | | |
| Under 16 years of age... ... ... ... | 3,279 | 2,167 | 5,446 | 3,265 | 1,942 | 5,207 |
| 16 years and above ... ... ... ... | 11,811 | 6,096 | 17,907 | 11,390 | 5,962 | 17,352 |
| Total: | | | | | | |
| Under 16 years of age... ... ... ... | 11,624 | 7,148 | 18,772 | 12,069 | 6,738 | 18,807 |
| 16 years and above ... ... ... ... | 68,445 | 48,549 | 116,994 | 69,600 | 46,515 | 116,115 |

From this table we learn that there was, in 1859 (1), a general decrease of the males of every class, with the exception of vagrants; and (2) an increase of the female thieves, except those under 16 years of age; but with regard to prostitutes, an increase both of the juvenile and the adult, making together 7·0 per cent.; yet the gross total does not vary more than is represented by an increase of 0·7 per cent. (p. vii.)

The *proportion of the criminal classes in the chief districts* is attempted to be shown by calculations based upon population, and with the following results—the districts being arranged in the order of criminality, commencing with the highest, and progressing to the lowest:—

| | Criminal Classes. |
|---|---|
| 1. *Seats of the Hardware Manufacture.*—Birmingham, Sheffield, and Wolverhampton | 7,685, or 1 in 54·4 |
| 2. *Towns depending upon Agricultural Districts.* — Ipswich, Exeter, Reading, Shrewsbury, Lincoln, Winchester, Hereford, and Bridgewater . . . . | 1,854, or 1 in 86·6 |
| 3. *Pleasure Towns.*—Brighton, Bath, Dover, Leamington, Gravesend, Scarborough, and Ramsgate . . . . . . | 2,265, or 1 in 87·4 |
| 4. *Commercial Ports.*—Liverpool, Bristol, Newcastle-on-Tyne, Kingston-on-Hull, Sunderland, Southampton, Swansea, Yarmouth, Tynemouth, and South Shields | 9,389, or 1 in 96·4 |
| 5. *The Rural Districts* . . . . . . | — 1 in 110·4 |
| *Eastern Counties.*—Essex, Norfolk, Suffolk, Lincoln . . . . . . | 10,407, or 1 in 122·0 |

| | Criminal Classes. |
|---|---|
| *South and South-Western Counties.*—Southhmptn, Wilts, Dorset, Somerset | 9,644, or 1 in 106·7 |
| *Midland Counties.*—Cambridge, Bedford, Northmptn, Hertford, Oxford, Bucks, Berks . . . . . . | 8,966, or 1 in 102·5 |
| 6. *Seats of the small and mixed Textile Fabrics.*—Norwich, Nottingham, Derby, Macclesfield, Coventry, Newcastle-under-Lyne, and Congleton . . . . | 2,208, or 1 in 119·4 |
| 7. *Seats of the Cotton and Linen Manufacture.*—Manchester, Preston, Salford, Bolton, Stockport, Oldham, Blackburn, Wigan, Staleybridge, and Ashton-under-Lyne . | 6,090, or 1 in 124·6 |
| 8. *Seats of the Woollen and Worsted Manufacture.*—Leeds, Bradford, Halifax, Rochdale, Huddersfield, and Kidderminster . | 2,779, or 1 in 137·0 |
| 9. *The Metropolis.*—Including an average radius of 15 miles round Charing Cross, and comprising the district of the Metropolitan and the City of London Police . | 13,120, or 1 in 194 |

These results correspond with those obtained from the returns of the previous year, with one exception. In 1858, the pleasure towns ranked after, not as in 1859 before, the commercial ports, in order of criminality. It is to be remarked also that, contrary to the usually received opinion, the metropolitan districts are in a marked degree least infested with the criminal classes. But, it is not to be forgotten that, as Mr. Redgrave remarks, "In the agricultural districts, the bad characters would be more readily known and traced by the police than when hidden in the population of towns, and their relative number is high, especially if the prostitutes, who congregate chiefly in the towns, are omitted from the calculation."

The distribution of prostitutes in the different districts is as follows, those only being included in this class who notoriously and obviously belong to it:—

| | Prostitutes. |
|---|---|
| 1. *Commercial Ports* . . . . | 5,221, or 1 in 173·4 |
| 2. *Pleasure Towns* . . . . . | 943, or 1 in 209·9 |
| 3. *Towns depending upon Agricultural Districts* . . . . . | 666, or 1 in 241·0 |
| 4. *Seats of the small and mixed Textile Fabrics* . . . . . . | 762, or 1 in 345·9 |
| 5. *The Metropolis* . . . . . . | 6,849, or 1 in 371·6 |
| 6. *Seats of the Hardware Manufacture* . | 922, or 1 in 453·5 |
| 7. *Seats of the Cotton and Linen Manufacture* . . . . | 1,616, or 1 in 469·7 |
| 8. *Seats of the Woollen and Worsted Manufacture* . . . | 681, or 1 in 559·2 |

| | | Prostitutes. |
|---|---|---|
| 9. *The Rural Districts* . . . . | —— | 1 in 1109·3 |
| Eastern Counties . . . . | 1·104, or | 1 in 1150·2 |
| South and South-Western Counties . | 1,394, or | 1 in 738·7 |
| Midland Counties . . . . | 639, or | 1 in 1439·0 |

The returns of *houses of bad character* show an increase of 4·6 per cent. upon the numbers of the preceding year. This was due chiefly to a larger number of public houses and beer shops of bad resort, but this may have arisen rather from the improved observation of the police than from any actual increase in the number of houses. The total numbers were :—

| | | |
|---|---|---|
| Houses of receivers of stolen goods . . . . | | 3,041 |
| Houses the resort of thieves and prostitutes, viz. :— | | |
| Public houses . . . . . . . | 2,811 | |
| Beer shops . . . . . . . . | 2,765 | |
| Coffee shops . . . . . . . . | 428 | |
| Other suspected houses . . . . . | 1,946 | |
| | —— | 7,950 |
| Brothels and houses of ill-fame . . . . . | | 7,991 |
| Tramps' lodging-houses . . . . . . | | 7,294 |
| Total houses of bad character . . . . | | 26,276 |

3. *Crimes committed and Apprehensions.*—Under this head, Mr. Redgrave remarks :—

"The crimes known and recorded by the police are of the class which are proceeded against in the criminal courts to the exclusion of the lesser offences ; and if it could be assumed that all such crimes are included in these returns, their numbers, when compared with the apprehensions which ensue, would be a successful proof of police vigilance. But though it may be concluded that crimes which from their atrocity or magnitude cause alarm, and hue and cry, will not fail to be known and recorded by the police, it cannot be supposed that the large amount of petty depredation which must result from the number of the criminal class already enumerated can be fairly represented by the 36,262 cases which appear in the returns under the wide definition of *Larceny*. Such a statement must rather be taken as a proof of how little accurate information is possessed of the extent of the pilfering and depredation which all the evidence in the returns tends to show must be successfully committed." (p. ix.)

Crime, it would appear, prevails chiefly in the long nights of the winter season, when there is also the greatest dearth of employment. The apprehensions amount to 52·1 per cent. of the crimes, and are proportionably 2·0 per cent. higher in the summer than winter season. The distribution of crime in the different seasons was as follows :—

| | Crimes committed. | Persons Apprehended. |
|---|---|---|
| October, November, and December . | 14,278 | 7,366 |
| January, February, and March . . | 13,999 | 7,113 |
| April, May, and June. . . . | 11,903 | 6,368 |
| July, August, and September . . | 11,838 | 6,272 |
| | 52,018 | 27,119 |

The returns represent the state of crime in the different police districts, but, looking only to some of the graver offences, we find that, of 95 murders, 12 were committed in the metropolitan police district; 19 in Lancashire; and 12 in Yorkshire. Concealing the birth of infants, with which infanticide is so closely allied, prevails in the rural districts; cases of rape and attempts to ravish were mostly in the same districts. Burglary and housebreaking are commonest in the country districts; robbery on the person chiefly occurs in the country districts, the metropolitan police district, Birmingham, Manchester, Liverpool, and Leeds. (p. x.)

The persons apprehended by the police were disposed of by the magistrates in the following manner:—"37·5 per cent. were at once liberated after a detention of probably only a few hours; 4·7 per cent. were discharged on finding bail to appear and take their trial; and 56·4 per cent. were committed to prison to await trial at the next sessions."

Of the pursuit of criminals the subsequent interesting and im portant results are stated:—

"In the offences against the person, 2579 crimes are recorded, out of which arose 2768 apprehensions, more than one person being frequently implicated in such offences, and 1908 commitments for trial, so that 73·9 per cent. of the cases were successfully pursued by the police, a very satisfactory proportion, making every allowance for the cases where more than one commitment ensues. In the violent offences against property, 4433 crimes are followed by 2204 apprehensions, and 1609 commitments for trial, or 36·3 per cent., and probably of these two classes of crimes the great proportion are known to the police and included in these returns. Next follow the ordinary cases of theft, embezzlement, fraud, offences unaccompanied by violence; these, as I have already stated, appear very inadequately represented by the 41,370 cases reported. They moreover led to only 18,738 apprehensions and 11,437 commitments for trial, not more than 27·6 per cent. of the offences recorded, which confirms the opinion that up to this time there exists a great impunity and long career in petty thefts, when unaccompanied by such acts of violence as create alarm and stimulate prompt information to the police to be followed by active pursuit."

4. *Summary Convictions.*—"The proceedings under the summary jurisdiction of justices are by information (except where persons are in the custody of the police, and the justices determine to deal with the charges summarily), and the accused appears on a summons obtained against him; if in such cases the justice does not convict, or if on conviction a fine is imposed and paid, the accused is not detained, and has never in fact been in custody, imprisonment therefore in all summary cases only ensues after conviction." In 1859, charges against 392,800 persons (310,690 males and 82,120 females) were determined summarily, of whom 257,810 (213,494 males and 44,316 females) were convicted. "It is remarkable," Mr. Redgrave states, "that throughout the criminal statistics the females, from no other apparent cause than greater leniency towards the sex, bear a much smaller proportion in the convictions than the males. On the above convictions this difference is nearly 15 per cent., the proportion being of the males 68·7, of the females 53·9." (p. xi.)

Of the number convicted, 57,284 were imprisoned (780 in reformatory schools, 37,366 under one month, and the remainder, with the exception of 71, under six months); 57,284 were fined; 162,204 whipped; and the rest were subjected to other punishments, delivered up to the army or navy, or ordered to find sureties or to enter into recognizance.

Of the offences determined summarily 37,339 are classed under the head of *stealing and attempts to steal;* 3650 were *malicious offences of damage and trespass;* 84,033 *assaults;* 8188 *offences against the Game Laws;* and of the remainder, we may note 89,903 *drunkenness and disorderly conduct;* 25,757 under the *Vagrant Laws;* 12,944 under the *Licensed Victuallers and Beer Acts;* 6341 under the *Weights and Measures Acts;* and 4553 *nuisances and offences against health.*

The number of persons proceeded against in 1859 shows a decrease of 2·7 per cent. upon the total proceeded against in the previous year, the convictions being 0·9 per cent. less.

5. *Character of the Accused.*—The class and character of the persons apprehended by the police, both those proceeded against on indictment and summarily, were as follows:—

| Characters. | Proceeded against on Indictment. | | Proceeded against Summarily. | | Total proceeded against. | | |
|---|---|---|---|---|---|---|---|
| | M. | F. | M. | F. | M. | F. | M. & F. |
| Known Thieves ... ... ... ... | 4,207 | 1,207 | 13,213 | 3,397 | 17,420 | 4,604 | 22,024 |
| Prostitutes... ... ... ... ... | — | 2,064 | — | 20,691 | — | 22,755 | 22,755 |
| Vagrants, Tramps, and others without visible means of subsistence ... ... ... ... | 529 | 151 | 11,695 | 4,026 | 12,224 | 4,177 | 16,401 |
| Suspicious characters ... ... | 5,378 | 1,261 | 38,374 | 6,550 | 43,752 | 7,811 | 51,563 |
| Habitual Drunkards (not under above heads) ... ... ... ... | 353 | 98 | 18,087 | 5,205 | 18,440 | 5,303 | 23,743 |
| Previous good Character... ... | 3,931 | 886 | 113,770 | 14,772 | 117,701 | 15,658 | 133,359 |
| Character unknown ... ... ... | 5,522 | 1,532 | 115,551 | 27,479 | 121,073 | 29,011 | 150,084 |
| Total ... ... ... ... ... | 19,920 | 7,199 | 310,690 | 82,120 | 330,610 | 89,319 | 419,929 |

Upon this table Mr. Redgrave remarks :—

"Comparing the number of the above classes proceeded against with the numbers reported to be at large, the police are shown to have pursued during the year 22,024 known thieves and depredators against 39,538 stated to be at large, or 55·7 per cent., and 22,755 prostitutes against 30,780 at large, no less than 73·8 per cent. This may be taken as a proof of the active agency of the police in the repression of the criminal classes." (p. xiii.)

This repressive agency of the police is shown more conspicuously by a statement of the commitments of known thieves in the town and country districts already referred to.

6. *Coroners' Returns.*—The inquests held in 1859 exceed in number those of 1858 (3·4 per cent.) and 1857, and they presented one noticeable fact, to wit, "the great increase of the number of females murdered"—exceeding by one-third the average of the three previous years. The number of inquests held last year, and the verdicts, are thus stated :—

| Verdict. | Males. | Females. | Total. |
|---|---|---|---|
| Murder | 89 | 115 | 204 |
| Manslaughter | 140 | 58 | 198 |
| Justifiable homicide | 17 | 6 | 23 |
| Suicide or self-murder | 910 | 330 | 1,240 |
| Accidental death | 7,081 | 2,160 | 9,241 |
| Injuries, causes unknown | 223 | 127 | 350 |
| Found dead | 1,873 | 1,044 | 2,917 |
| Natural death :— | | | |
| From excessive drinking | 206 | 100 | 306 |
| Disease aggravated by neglect | 40 | 53 | 93 |
| Want, cold, exposure, &c. | 106 | 56 | 162 |
| Other causes | 3,409 | 2,388 | 5,797 |
| Total | 14,094 | 6,437 | 20,531 |

Of the subjects of the foregoing inquests 52·5 per cent. were adults, and nearly one-third (31·3) per cent. females. Infants of 7 years of age and under were the subjects of 5605 inquests, and children under 16 and above 7 years, of 1784.

The total cost of the inquests in 1859 was 60,920*l.* 16*s.* 6*d.*, the average expense of each inquest being 2*l.* 19*s.* 4*d.*

7. *Present State of Crime.*—A comparative view of the numbers of commitments over a series of years (furnished by the records of trials and punishments in the "Criminal Tables" since 1810) indicates an arrest in the onward progress of crime, and "affords the hope of a permanent improvement." In the past year the commitments were 6·6 per cent. less than in 1858, and 17·7 per cent. less than in 1857. This decrease was very general, reaching all the English counties except Bedford, Cornwall, Durham,

Middlesex, Northampton, Rutland, Southampton, Westmoreland, and in each of these counties the commitments were still less than in 1857. There was a decrease of 5·8 per cent. in the commitments for *offences against the person*, although murder and attempts to murder, concealing the births of infants, and bigamy formed an exception to the rest of this class, showing an increase, but only in an unimportant degree. Stabbing, manslaughter, unnatural offences, and rape and attempts to ravish, had diminished, the latter considerably. In 1858, there was a large decrease of commitments for *offences against the person*. Last year a further decrease of 15·8 per cent. occurred, including the chief crimes of burglary, house-breaking, and robbery. A greater or less decrease of the commitments in the remaining classes of offences also occurred, with the exception of malicious ones against property, in which there was a slight increase.

But the present state of crime will be better seen by a further retrospect with which Mr. Redgrave furnishes us, first observing that:—

"There are distinctly two classes of offences, one of which springs from the state of the general community, and is of singularly uniform recurrence; the other from a separate criminal class, from time to time increased or diminished in number by external circumstances, as the price of food, the state of employment, and again more directly by changes in the police and the criminal laws, by which the class is repressed. It has also been found that in those years when the tendency to petty thefts and frauds is lessened by abundant employment for labour and cheap food, assaults and other minor offences against the person are stimulated, probably by the increased means of obtaining intoxicating liquors, which such periods afford." (p. xv.)

Mr. Redgrave then goes on to state :—

"The data exist for an accurate comparison of the state of crime on the commitments of the last thirty years, and selecting those crimes for which no disturbing changes have been made in the law, except so far as refers to a great amelioration of the punishments, the following results are obtained. First, with regard to those crimes which may be held largely to mark the character of the community.

| Commitments in each five Years. | 1859-55. | 1854-50. | 1849-45. | 1844-40. | 1839-35. | 1834-30. |
|---|---|---|---|---|---|---|
| Murder . . . | 345 | 348 | 365 | 347 | 315 | 326 |
| Stabbing, wounding, &c. with intent to murder or do bodily harm . | 1505 | 1249 | 1173 | 1157 | 739 | 605 |
| Manslaughter . . | 1144 | 1144 | 980 | 1050 | 1024 | 912 |
| Rape and attempts to ravish . . . | 1239 | 1395 | 1263 | 1221 | 973 | 837 |
| Bigamy . . . | 449 | 404 | 399 | 354 | 215 | 186 |

"In the thirty years over which this comparison extends, the population cannot be estimated to have increased less than forty per cent.

(which would account for a corresponding increase of crime), and property probably in a much greater ratio. It is satisfactory, therefore, to point out that there has been no increase during that period in the commitments for murder; but in the attempts to murder, stabbing, wounding, &c., there has been a progressive increase, which showed itself in a marked degree in 1837, on the extensive abolition of capital punishments which was then effected. In manslaughter the increase has been small, not amounting to one-half the rate of increase of the population. The commitments for rape and attempts to ravish, which on the abolition of the capital punishment in 1841 at once attained a higher rate, and have since been without important change, increased in the last period of fifteen years 28·5 per cent., and in bigamy a large progressive increase marks each quinquennial period. These crimes are chiefly those in which the detective agency of the increased police establishments would be brought to bear rather than their powers of prevention, and there can be no doubt that the increase described may in some considerable degree be attributed to the greater ratio of detection." (p. xvi.)

On a comparison for the same period of the crimes which may be attributed to the existence of a criminal class, the results are by no means unfavourable.

"Taking the worst offences which arise from the criminal class, and are chiefly committed by the old offenders, it is shown that for the last fifteen years the commitments for burglary and house-breaking have been without any sensible variation, and that for robbery on the person the increase, comparing the first with the last fifteen years, has not exceeded 13·7 per cent.; but for horse, sheep, and cattle stealing in the same period, there has been an absolute *decrease* in the commitments, which has been continuous, and reaches 31·0 per cent. These are all crimes in which the agency of the police would be immediately felt, and particularly in the repression of horse, sheep, and cattle stealing. The whole tendency of crime has been, for some years, to the diminution of offences of violence, and the increase of offences of planned theft and fraud—skill in crime has succeeded violence. This is apparent in the above commitments, the increase of all the latter class is most marked. It is gratifying again to notice the entire absence of all offences of a seditious or treasonable character for above ten years." (p. xvi.)

8. *Results of Trials.*—Of the 16,674 persons committed in 1859, 4175 were acquitted and discharged; 15 were acquitted on the ground of insanity, and 14 found insane, making a total of 29 detained as insane; 52 were sentenced to death; 2170 to penal servitude; 10,060 to imprisonment; and 188 to whipping, fine, &c., forming a total of 12,470 convicted.

A comparison of the sentences pronounced during the last ten years, shows that "the decrease of commitments has been concurrent with a diminished severity of the punishments." Transportation as a sentence has ceased, but the power to remove convicts to the penal colonies is reserved by the abolishing statute (20 and 21 Vict.) Capital convictions do not average 55 yearly; and under 75 will probably be the average sentenced to penal servitude for life.

"In above 80 per cent. of the convictions the sentence is one of imprisonment, and rarely for a term exceeding one year." Last year, 52 persons were sentenced to death, and 9 executed; of the remainder, 41 were subjected to penal servitude, the majority for long periods and 13 for life, 1 was sent to a reformatory for five years, and 1 received a free pardon. In 1829, 1385 were sentenced to death and 74 executed, "51 of whom were convicted of crimes not now capital."

The costs of prosecutions for the year terminating the 30th June, 1858 (the last return), amounted to 166,229*l.* 1*s.* 9*d.*

9. *Crime in France and England.*—A brief comparison of the status of crime in France and England, based upon the latest official returns of both countries, leads to one or two highly interesting conclusions. It would appear that the most serious offences against the person are nearly twice as prevalent in France as in England, the relative population being considered, whereas it is probable that crimes against property are slightly in excess, and simple thefts and frauds markedly in excess in England.

A comparison of the number of offences proceeded against in the different courts of the two kingdoms is slightly in favour of England. Thus the total number of persons proceeded against in France, in 1857, amounted to 771,374 = 213·7 in every 10,000 population; in England, in 1859, the total number was 409,484 = 209·5 in every 10,000 population.*

Assuming that the total amount of crime in both countries, relative to population, is about the same, the greater tendency to the graver offences in the one country is a fact of weighty interest to the practical psychologist. The importance of the fact will be best understood by quoting Mr. Redgrave's comparative statement of the crimes murder and rape in the two countries:—

| France, 1857. | | England, 1859. | |
|---|---|---|---|
| Meurtre | 59 | Murder | 70 |
| Tentative de | 49 | Attempts to murder, attended with dangerous bodily injuries | 22 |
| Assassinat | 155 | Attempts to murder, not attended with dangerous bodily injuries | 11 |
| Tentative d' | 88 | | |
| Parracide et tentative de | 15 | | |
| Infanticide et tentative d' | 246 | | |
| Empoisonnement | 24 | | |
| Tentative d' | 23 | | |
| Total murder and attempts to murder | 659 | Total murder and attempts to murder | 103 |

* Estimated population of France in 1857, 36,090,911; census of 1856, population 36,039,364. Estimated population of England, 1859, 19,542,785; census of 1851, population 17,927,609.

FRANCE, 1857.

| | |
|---|---|
| Attentat à la pudeur avec violences sur des adultes, sans circonstances aggravantes . . . . | 73 |
| Viol et attentat à la pudeur avec circonstances aggravantes sur des adultes . . . . . . . . | 130 |
| Attentat sans violences sur des enfans au-dessous de onze ans, sans circonstances aggravantes. | 300 |
| Viol et attentat à la pudeur avec violences sur des enfans de moins de 15 ans, ou sans violences, mais avec d'autres circonstances aggravantes . . . . . . . | 332 |
| Attentat à la pudeur par un individu de moins de 16 ans (délit) | 55 |
| Total rape and attempts to ravish . . . . . . . . | 890 |
| Avortement et tentatives de . . | 104 |

ENGLAND, 1859.

| | |
|---|---|
| Rape, and carnally abusing girls under the age of ten years . . | 132 |
| Assaults with intent to ravish and carnally abuse . . . . . . . | 131 |
| Carnally abusing girls between the age of 10 and 12 years . . | 11 |
| Total rape and attempts to ravish . . . . . . . . . . | 274 |
| Attempts to procure miscarriage . | 7 |

The frequency of parricide and infanticide, as well as the excess of the grossest offences against morality, in the French records is very remarkable. A further comparison cannot well be instituted between the graver offences against the person in the two countries. The manslaughter and malicious stabbing of our law do not precisely correspond with the homicide and *blessures et coups* of the French code. The latter, however, so greatly exceed the seemingly-like class of cases in England, that it is probable the French terms include cases which in this country would be proceeded against as assaults only.

10. *Commitments to Prison.—Prisons and Prisoners.*—The commitments to prison, in 1859, were 9·0 per cent. less than in 1858 (the commitments of that year being 1·7 per cent. less than in 1857), and there was, for the first time, a marked decrease in the female commitments. "These," Mr. Redgrave states, "had increased from year to year till 1857, when they reached their maximum, having added 35·8 per cent. to their numbers since 1847. Separating, however, the more strictly criminal commitments, those for trial and on summary conviction, it is shown, on a comparison of the two last periods of five years, that this class of the commitments decreased 2·9 per cent., while the total commitments *increased* 4·9 per cent." (p. xxiii.)

The fact of a progressive decrease in the number of commitments to prison "in face of the large body of police lately added to existing establishments, and the concurrent almost complete abandonment of transportation to the colonies must be taken as a conclusive proof of the decrease of crime." (p. xxiii.)

The *recommittals* (38,428) last year were 3152 less than in

1858 (41,580), their proportion being to the total commitments 27·5 per cent. This proportion was 29·8 per cent. in 1858, and 29·7 per cent. in 1857. These figures, however, cannot be regarded as representing the full amount of recommittals; neither, on the other hand, is it to be supposed that all those, who, after being discharged from prison, are lost sight of by the police, are reformed. If, on enlargement, a *quondam* criminal seeks new haunts and recommences there a course of crime, should he again come within the grasp of the law, which sooner or later is pretty certain to happen, his previous committal often cannot be recorded against him, his former history being generally unknown to the police of the locality.

The proportion of *juvenile commitments*, that is to say, those under 16 years of age, reached its maximum in 1856, and was then 12·3 per cent. on the total commitments. In 1857, the proportion was 10·0 per cent.; in 1858, 8·7 per cent.; and in 1859, 8·8 per cent. The number of commitments, last year, under 12 years of age, was 1378 (1176 males and 202 females); aged 12 and under 16 years, 7535 (6406 males and 1129 females).

Of the total number of individuals committed to prison in 1859, 77·9 per cent. were *born* in England; 2·6 in Wales; 2·1 Scotland; 14·2 Ireland; 0·5 the Colonies and East Indies; and 1·6 foreign countries; 1·1 per cent. not being ascertained.

The *state of instruction* of the committed was as follows:—35·7 per cent. could not either read or write; 58·8 per cent. could read, or read and write imperfectly; 4·3 per cent. could read and write well; 0·3 per cent. had inferior instruction; and of 0·9 per cent. the instruction was not ascertained. These figures, on being compared with those of previous years, show a tendency to an increase of prisoners who are able to read and write imperfectly.

The *occupations* of the committed may be thus summed up:—19·4 per cent. had no occupation; 4·1 were domestic servants; 42·4 are classed as labourers, charwomen, and needle-women; 4·5 were factory workers; 19·6 were mechanics and skilled labourers; 0·1 foremen and overlookers of labour; 1·1 shopmen, shopwomen, clerks, &c.; 3·1 shopkeepers and dealers; 0·3 professional employments; 4·4 sailors, mariners, and soldiers; 1·0 occupations not ascertained. This return, as compared with the similar one of the previous year, shows a decrease in the total number of prisoners recorded as of "no occupation," although, it is to be remarked, that there was an increase of females, probably prostitutes, under this head.

At the commencement of 1859, the number of prisoners confined in county and borough gaols amounted to 17,920. During the year, 126,861 persons were committed, and the removals between local prisons numbered 3514, giving 148,295 as the total amount of prisoners in 1859. Of these, 8991 were removed to

Government and other prisons, 936 (766 males and 170 females) to reformatory schools, and 124 (96 males and 28 females) to lunatic asylums; 163 were discharged on pardon or commutation of sentence, 6 on ticket-of-leave, and 122,316 on the termination of sentence or commitment, 6 escaped, 9 committed suicide, 160 died, and 10 were executed—15,574 remaining in prison at the end of the year.

The decrease of commitments, as previously stated, was, in 1859, 9·0 per cent.; but the decrease in the number remaining in prison at the end of the year was 13·0 per cent.; a difference to be accounted for by the lesser duration of the sentences, added to some increase in the proportion removed and discharged.

Our prisons are constructed to contain 25,858 prisoners (20,659 males and 5199 females); the greatest number confined at any one time last year was 20,693 (16,034 males and 4659 females); while the daily average was 16,709 (12,998 males and 3711 females). Many of our prisons, notwithstanding the large margin apparent in the summary, are still inadequate to their average commitments.

There is a tendency to an increase of *sickness* in the prisons, chiefly manifested in cases of slight indisposition. The infirmary cases and death-rate keeps pace, however, with the decrease of commitments. The cases of insanity among prisoners amounted, in 1859, to 129 (98 males and 31 females); the average annual number of cases in the five years, 1849-53, being 106; and in 1854-59, 132.

The returns of *prison punishments* show a greater decrease than the amount of prison population, and chiefly in the severer punishments inflicted by order of the magistrates. "The offences which the keepers of prisons may punish are defined, and the punishments are restricted to close confinement in a solitary or refractory cell, with bread and water only, for a term not exceeding three days." In 1859, whipping was had recourse to in 108 instances: irons or handcuffs, in 88; solitary or dark cells, in 11,002; stoppage of diet, in 35,065; and other punishments, in 1441—punishment being thus inflicted in 47,704 instances, the distribution being to the male prisoners, 39,172; to the female, 8532. Whipping was confined solely to the male prisoners.

The cost of the county and borough prisons in 1859 amounted to 493,747*l.* 6*s.* 7*d.*, the average annual cost of each prisoner being 29*l.* 10*s.* 11*d.*, or, excluding the extraordinary charges for building and fittings, 24*l.* 5*s.* 11*d.* The profit arising from prisoners' labour was 25,410*l.* 7*s.* 9*d.*; vagrants' money applied to maintenance, and other small contingent receipts, raised the prison receipts to 25,513*l.* 1*s.* 6*d.* The remainder of the funds were supplied from the following sources: Local rates and funds, 364,816*l.* 13*s.* 6*d.*; public revenues, 99,417*l.* 11*s.* 7*d.*

The *convict prisons*, which are entirely appropriated to prisoners sentenced to penal servitude, and which have superseded the hulks, are—*Pentonville* and *Millbank*, for prisoners undergoing the first stage of discipline in separate confinement, both males and females; *Portsmouth*, *Portland*, and *Chatham*, for male prisoners undergoing the second stage of discipline and industrial training on public works; *Dartmoor*, for male invalids capable only of light labour on the land; *Lewes*, temporarily used for confirmed invalid male prisoners, and to be given up on the completion of the new invalid prisons now erecting at Woking; *Parkhurst*, Isle of Wight, the reformatory for convicted boys; *Brixton*, exclusively for female prisoners under the second stage of discipline; and the *Fulham Refuge*, also exclusively for female prisoners under instruction, and employed in laundry and other useful domestic work.

These prisons are under the control of the Secretary of State for the Home Department, and their management is superintended by the Chairman and Directors of Convict Prisons. Additional accommodation for convicts is also provided, at the charge of Government, in two county prisons.

At the commencement of 1859, 7628 convicts were undergoing punishment; and 2755 were received from county and borough prisons during the year, making a total of 10,383 (8922 males, and 1461 females). Of these, 360 were removed to the colonies (224 to Western Australia, and 140 to Gibraltar), 1 was removed to a county gaol, 1 to a reformatory, and 38 were sent to lunatic asylums; 1747 were discharged on termination of sentence, 252 on tickets-of-leave, 24 on commutation of sentence, and 13 on pardon; 87 died, 1 committed suicide, and 3 escaped, while 7852 remained in prison at the end of the year—the daily average of convict prisoners during the year having been 7749.

The increased number of convicts at the termination, as compared with the commencement of the year, arose chiefly from the greater number of females under detention. The daily average shows a decrease of 14·1 per cent. on the preceding year; and the number of convicts sent to the colonies, as compared with two preceding years was greatly diminished. Thus, in 1857, 1032 were sent abroad; in 1858, 1390; in 1859, 364 only.

On *transportation*, and particularly on the transportation of female convicts, also on *tickets-of-leave*, Mr. Redgrave makes the following remarks:—

"It is probable that owing to the greater difficulties and restrictions which now attend transportation, diminished numbers will for the future be removed to a penal colony. There has been for some time no suitable means of providing for the transportation of *female* convicts, which, added to the increase of the offenders of that sex, and the admitted greater difficulty of their reformation, will sufficiently account for the increased numbers in the convict prisons, as well as in the county and borough prisons, where the unusually large proportion of the repeated recommittals of females show that no proper discipline has yet been found for the lost characters of this class.

"The conditions have nearly disappeared which on the sudden restriction of the means of transportation to a penal colony necessitated a large discharge of convicts at home and led to the adoption of the licence or ticket-of-leave system. It is probable that in the limited number of such discharges now granted the licence will prove an additional safeguard to the public and a wholesome restraint to the discharged convict. These discharges were, in 1856, 2892; in 1857, 922; in 1858, 312; and in 1859, 252 only." (p. xxxi.)

The *sickness* among the convicts needs no remark, except that the females suffered much less than the males, 350 cases of serious illness being recorded among the former, while 6663 occurred among the latter: the cases of insanity, 31 males, 9 females. The *punishments* barely averaged one a-day, and were chiefly of the same character as those noted in the county and borough prisons. The instances to which whipping was had recourse, were less than half the average number.

The total cost of the convict prisons amounted to 247,716*l.* 14*s.* 4*d.*; the average cost of each convict, 31*l.* 19*s.* 3*d.* This return is exclusive of gratuities paid to convicts on discharge, with allowance for their clothing and travelling, which average about 20,000*l.* additional. The monetary value of convict labour, although it must largely reduce the costs involved in their restraint, cannot readily be estimated.

The prisoners in the *reformatory schools* during 1859, amounted to 3014 (2488 males, and 526 females), of whom 922 had been committed during the year, 472 of these cases having been previously under restraint. The number of prisoners discharged in 1859 was 441, and there remained under detention, at the end of the year, 2426.

It is most gratifying to find, and at the same time it is a most hopeful indication of the good and permanent effect of these schools, that the decrease of crime in 1859 was contemporaneous with an increase of the numbers discharged from them.

"It seemed reasonable," writes Mr. Redgrave, "on a first experience to impute the decreased proportion of recommittals to the large numbers who were undergoing an unusually lengthened detention under the new reformatory discipline. But it is the more satisfactory now to show that the previous commitments, instead of increasing with the numbers discharged, have actually largely diminished, thus:—

| | | | |
|---|---|---|---|
| "Discharged, | 1859 . . . 441 | Previously committed, | 1859 . . . 472 |
| " | 1858 . . . 299 | " " | 1858 . . . 496 |
| " | 1857 . . . 108 | " " | 1857 . . . 663" |

(p. xxxiv).

The total cost of the reformatory schools (defrayed from the public revenues at the rate of 7*s.* each prisoner) was, last year, 38,853*l.* 1*s.* 3*d.* The total sum received from the parents in diminution of this charge, 1594*l.* 0*s.* 8*d.*

Of the *industrial schools* (19 in number, 11 being in the metropolis) to which children under 14 years of age can be directly committed, without any intervening imprisonment in gaol, little can yet be said, the Act governing them having hitherto been comparatively inoperative.

11. *Criminal Lunatics.*—A return of the criminal lunatics under detention completes Mr. Redgrave's Report on Police, Criminal Proceedings, and Prisons.

The number of criminal lunatics under detention at the commencement of 1859, was 684 (527 males and 157 females). During the year there were committed, 200 (162 males and 38 females), and received from other asylums 17 (14 males and 3 females), making a total of 901 lunatics under detention in the year. Of these, 43 died, 1 committed suicide, 4 escaped, 54 (36 males and 18 females) were discharged on becoming sane, 16 were removed sane, for trial or punishment, and 54 were removed to other asylums, leaving 729 (569 males and 160 females) under detention at the termination of the year.

"The numbers remaining under detention," Mr. Redgrave remarks, "form a further increase in 1859, the numbers in the former years having been, in 1858, 686; in 1857, 618; in 1856, 597; the increase being probably more owing to the greater vigilance exercised by the increased police forces, in providing for the safe detention of insane persons, than to their increase; but as their detention has no limit, the tendency must be to increased numbers." (p. xxxvi.)

The offences for which the foregoing lunatics were placed in custody (correctness of definition, however, not being always practicable) were as follows:—

| Offences. | Males. | Females. | Total. | Offences. | Males. | Females. | Total. |
|---|---|---|---|---|---|---|---|
| Murder ... ... ... ... ... ... | 95 | 44 | 139 | Larceny and petty thefts ... | 150 | 65 | 215 |
| Attempts to murder, maim, stabbing, &c.... ... ... ... | 81 | 8 | 89 | Frauds and embezzlements ... | 5 | — | 5 |
| Concealing birth and infanticide ... ... ... ... ... | — | 2 | 2 | Receiving stolen goods ... ... | 2 | 2 | 4 |
| Manslaughter ... ... ... ... | 4 | 2 | 6 | Arson and malicious burning | 35 | 5 | 40 |
| Rape ... ... ... ... ... ... | 4 | — | 4 | Wilful damage, and other malicious offences ... ... ... | 10 | 8 | 18 |
| Assault, with intent to ravish | 7 | — | 7 | Forgery ... ... ... ... ... | 2 | — | 2 |
| Unnatural offences ... ... ... | 10 | — | 10 | Uttering counterfeit coin ... | 7 | 1 | 8 |
| Treasonable and seditious practices ... ... ... ... | 2 | — | 2 | Riot and breach of the peace . | 33 | 7 | 40 |
| Assaults ... ... ... ... ... ... | 43 | 4 | 47 | Under the vagrant laws... ... | 34 | 14 | 48 |
| Indecent exposure of the person ... ... ... ... ... ... | 6 | — | 6 | Dangerous persons at large ... | 2 | — | 2 |
| Burglary and housebreaking . | 41 | 2 | 43 | Insane, wandering abroad without control ... ... ... | 1 | — | 1 |
| Robbery on the highway, &c. | 10 | — | 10 | Deserters from the army and navy ... ... ... ... ... ... | 6 | — | 6 |
| Sheep-stealing ... ... ... ... | 9 | — | 9 | Other offences ... ... ... ... | 79 | 24 | 103 |
| Horse-stealing ... ... ... ... | 8 | — | 8 | Total... ... ... ... | 686 | 188 | 874 |

The period of detention to which these lunatics had been subjected at the time of the return, is thus recorded:—

| | |
|---|---|
| One year and under . . . | 219 |
| Two years and above one . . . | 123 |
| Three years and above two . . | 116 |
| Five years and above three . . | 116 |
| Ten years and above five . . . | 118 |
| Fifteen years and above ten . . | 60 |
| Twenty years and above fifteen . . | 40 |
| Above twenty . . . . | 32 |

Of the 132 lunatics who have been detained upwards of ten years, 53 are under custody for murder and attempts to murder, maiming, or stabbing. Nearly one-third (247) of the total number of criminal lunatics, according to the table of offences, have been placed in confinement for grave offences against the person.

The total charge for the detention of criminal lunatics in 1859 was 23,376*l.* 16*s.* 5*d.*; of which 1657*l.* 13*s.* 9*d.* came from private funds.

From Mr. Redgrave's report on the proceedings of the civil courts, we shall only note the apparent *causes of bankruptcy* in 1859, adding also the summary of these causes for 1858:—

| | 1859. | 1858. |
|---|---|---|
| Reckless and unsound speculations, excessive trading . . . . . . . | 295 cases. | 457 cases. |
| Interest, discounts, accommodation bills, suretyship . . . . . . | 124 „ | 145 „ |
| Incompetence, neglect, personal extravagance | 323 „ | 432 „ |
| Unavoidable misfortunes . . . . | 145 „ | 197 „ |

In terminating this outline of the Status of Crime in 1859, we have to record that, with the present number of *The Judicial Statistics,* Mr. Redgrave's duties as the compiler of that work terminate. It is not, however, solely as the compiler of these important returns that Mr. Redgrave's relation to them is to be regarded. He has, indeed, a claim to much higher regard upon our part; for to his efforts, voluntarily undertaken and voluntarily carried out, we are chiefly indebted for the elaborate scheme of which we are now beginning to reap the first fruits. It is impossible, without an examination of the different returns upon which Mr. Redgrave's reports are based, to form a right idea of their comprehensiveness, and of the labour involved in securing the co-operation necessary to obtain the careful registration and periodical report of the facts upon which those returns are founded. It was only by long-continued and arduous efforts that Mr. Redgrave ultimately succeeded in overcoming the many disheartening difficulties which lay in his way; and now that his statistical scheme has been matured, and everything put in right train for its future working, we hope that no petty and mistaken economy on the part of Government will interfere with its being permanently carried out. The value of such a scheme of Judicial Statistics to the Legislature has been sufficiently insisted upon by Lord Brougham;* to the political and social economist its importance can hardly be questioned for a moment; to the philanthropist, the moralist, or the psychologist, *per se*, it offers a sound basis for the practical study of the general, as contradistinguished from the individual, phenomena of crime, the advantages of which cannot be exaggerated; to all persons these statistics must possess that high interest which is inseparable from an accurate knowledge of the amount and character of the overt villany which corrodes the nation, and of the influence of those means which are especially put in force for its repression.

For ourselves, while deeply regretting that Mr. Redgrave should find it requisite to retire from the active control of the Judicial Statistics, we would express our admiration of, and tender our thanks for, the great work he has effected for the nation—a work which, to his honour and our advantage, we trust will from henceforth have a permanent place among the annual records of Government.

---

### Art. IV.—THE MODERN DRAMA:

* "Full and minute statistical details are to the lawgiver, as the chart, the compass, and the lead to the navigator."—*Speech, House of Lords, March,* 1856.

